# Glucocerebrosidase Gene Mutations Associated with Parkinson's Disease: A Meta-Analysis in a Chinese population

**DOI:** 10.1371/journal.pone.0115747

**Published:** 2014-12-23

**Authors:** Jia Chen, Wei Li, Tao Zhang, Yan-jiang Wang, Xiao-jiang Jiang, Zhi-qiang Xu

**Affiliations:** 1 Department of Neurology, Daping Hospital, Third Military Medical University, 10 Changjiang Branch Road, Yuzhong District, Chongqing, 400042, PR China; 2 Department of Neurology, PLA 123 Hospital, 1052 Yanshan Road, Yuhui District, Bengbu, 233000, PR China; Duke University, United States of America

## Abstract

Mutations of glucocerebrosidase (GBA) confer susceptibility to Parkinson's disease in several ethnical populations, with a high incidence especially in the Ashkenazi Jewish population. Although there are several studies that have investigated a similar association in a Chinese population, small sample sizes and few positive outcomes have made it difficult to obtain conclusive results from these individual studies. Therefore, the present study used a meta-analysis approach, pooling the appropriate data from published studies to investigate the association of GBA mutations and Parkinson's disease in a Chinese population. Nine studies containing 6536 Chinese subjects (3438 cases and 3098 healthy controls) and examining the GBA mutations of L444P, N370S and several other mutations were included. Review Manager 5.2 software was applied to analyze the pooled odds ratios (ORs) and 95% confidence intervals (CIs). The results showed a significant association of Parkinson's disease risk with overall GBA mutations (OR = 6.34, 95% CI = 3.77–10.68, p<0.00001), and with the subgroup of L444P mutation (OR = 11.68, 95% CI = 5.23–26.06, p<0.00001). No such association was observed for the subgroup with N370S mutation or other mutations, in part because of the small sample size or rare events. Thus, for the rare occurrence of GBA mutations, studies with larger sample size are necessary to minimize the sampling error and to obtain convincing results.

## Introduction

Parkinson disease (PD), which ranks second only to Alzheimer's disease among neurodegenerative disorders, is characterized pathologically by the degeneration of dopaminergic neurons in nigrostriatal system and clinically by insidiously progressive movement impairments, such as rigidity, bradykinesia, impaired balance and tremor at rest [1]. In addition to aging and environmental factors, genetic risk is also considered as a critical factor for PD, including mutations in SNCA, LRRK2 and MAPT genes and other genomic loci, which have been identified as susceptibility genes for familial and common sporadic forms of PD [2].

Mutations of glucocerebrosidase (GBA) gene which located at chromosome 1q21 and encoding the enzyme glucocerebrosidase [3], have been significantly associated with PD susceptibility in Ashkenazi Jewish patients [4]. Subsequent to this finding, further evidence for this relationship was provided in non-Ashkenazi Jewish population, although some studies reported negative results [5], [6]. As an increased incidence of GBA mutations has been detected in brain samples from patients with PD [7], [8], it is necessary to know whether and how much GBA mutations confer susceptibility to PD. Among the GBA mutations, L444P and N370S heterozygotes are the most common variants, and patients with PD are five times more likely to carry these mutations than those without PD [9]. Several studies have investigated the relationship between GBA mutations and PD risk in various populations worldwide, including Portuguese, Brazilian, Canadian and Korean populations [10]–[13]. In Chinese population, there are several studies that have examined the association, however, it has not been fully elucidated. Therefore, to examine the relationship between GBA mutations and PD risk within Chinese population, we reviewed the relevant studies, determined the most studied mutations, and performed a meta-analysis.

## Materials and Methods

### Literature search

This study was conducted according to the Preferred Reporting Items for Systematic Reviews and Meta-analyses (PRISMA) criteria. Electronic databases, such as PubMed, Embase, Cochrane Library and PDgene, were systemically searched for eligible records, with no lower date limit but an upper limit of February 20, 2014. A combination of key words was used in the search strategy, such as “glucocerebrosidase” or “GBA”, “polymorphism” or “variant” or “mutation”, “Parkinson's disease” or “PD” and “Chinese”. The references within relevant reviews were manually examined to avoid missing eligible studies. The language was not restricted in the search.

### Inclusion and exclusion criteria

Studies included in the meta-analysis met the following criteria: (1) investigated the association between GBA mutations and PD in a Chinese population; (2) contained full text within the article; (3) contained sufficient data to calculate the odds ratio (OR), confidence interval (CI) and p-value; (4) specified genotyping methods or provided appropriate references; and 5) The diagnosis of PD should be based on established clinical evidence (bradykinesia and at least one of the following: muscular rigidity, resting tremor and postural instability), or meet UK PDS Brain Bank Clinical Diagnostic Criteria [14] or other recommended diagnostic criteria. People with suspected PD should be referred to a neurologist or movement disorder specialist to confirm diagnosis. The following exclusion criteria were used: (1) systemic reviews; (2) study that was not designed as a case-control pattern, or with no healthy controls; and (3) repeated or overlapping publications. In case of any missing details, we contact the authors by e-mail. When a study was reported in other publication or had an updated version, only the latest study was included in the present meta-analysis. Two investigators independently screened the titles, abstracts, and full texts to determine the suitability of the study for inclusion in this meta-analysis. Their results were compared and disagreements were resolved by consensus. The inter-rater reliability was determined after completing the selection process.

### Data extraction

Information was extracted from all included publications by two independent reviewers using a predesigned data extraction form. Disagreements were resolved by consensus. If the two reviewers could not reach a consensus, another reviewer was consulted. The following information was collected from each article: first author, publication date, study region, number of cases and controls, and the number of GBA mutations in PD cases and in controls.

### Statistical analyses

Because the frequency of GBA mutations is low, the Hardy–Weinberg equilibrium [15] for the control group was not tested. The strength of the association between GBA mutations and risk of PD was measured using the OR with the corresponding 95% CI for each study. The combined ORs were calculated. The chi-squared (χ^2^) test was used to assess the heterogeneity across studies, and *I^2^* statistics were calculated to quantify the proportion of total variation due to the heterogeneity. The ORs were pooled with a fixed effects model using the Mantel–Haenszel approach when no heterogeneity was observed among the studies [16]. Otherwise, a random effects model was adopted [17]. The strength of agreement between reviewers for their study selections was evaluated using the Kappa statistic [18]. A publication bias analysis was not performed for the limited number of studies. Two authors independently performed the statistical analyses and obtained the same results. Statistical analyses were conducted using Review Manager 5.2 software. For all tests, p-values less than 0.05 were considered statistically significant.

## Results

### Literature selection and study characteristics

**Figure 1:**
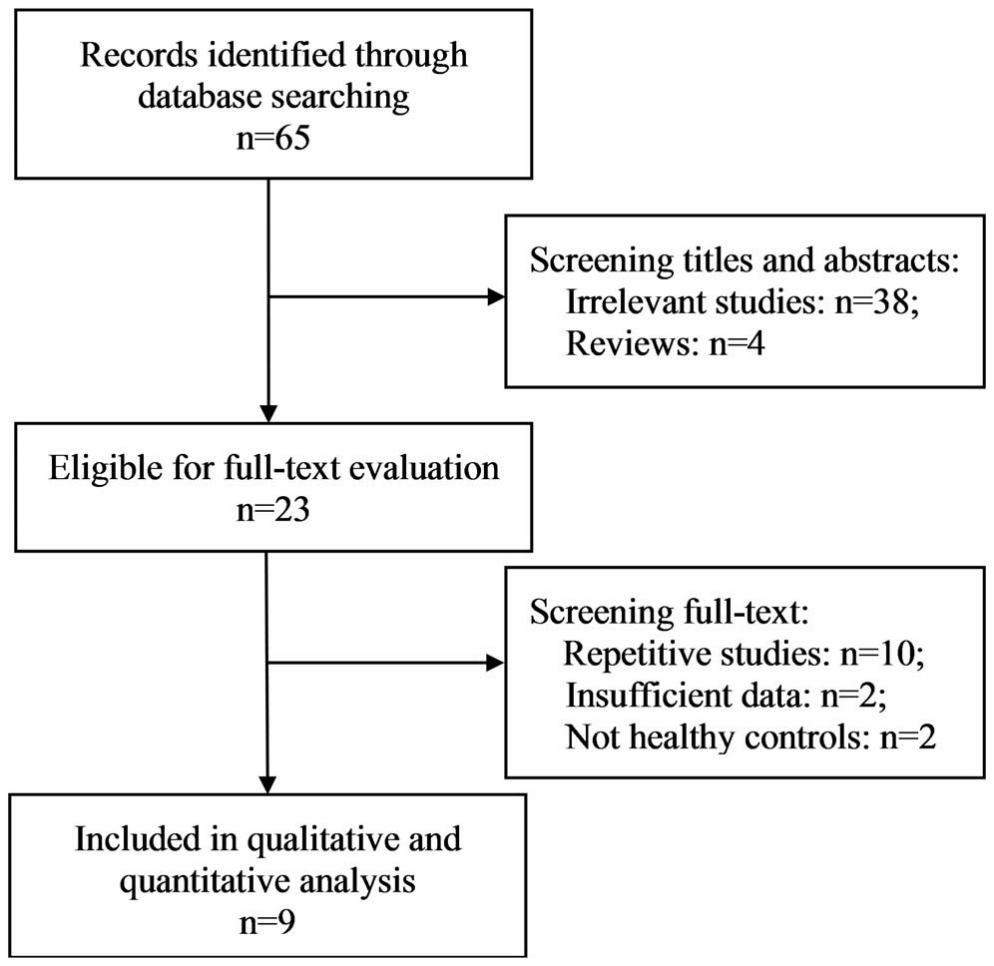
Flowchart showing the study selection.

Fig. 1 shows the detailed selection procedure. A total of 65 records were retrieved from online databases. After screening the titles and abstracts, 42 records were excluded. There were 11 records excluded for repeated studies or no interested outcome, and three records excluded for insufficient data or no healthy controls. Finally, nine publications were qualified with detailed data [19]–[27], including 3438 cases and 3098 controls. Among these records, one publication [21] contained several populations, but only Chinese population was used in the present analysis. The inter-rater reliability for the selection of studies included in the meta-analysis was nearly perfect, with Kappa values of 0.95 for the selection of titles and abstracts, and 0.89 for the selection of full texts. The characteristics of the studies are shown in Table 1, and the distribution of the GBA mutations studied in Chinese population is shown in Table 2. Among the listed mutations, L444P and N370S were the most studied mutations. Other mutation loci, such as Rec*Nci*I, R120W, D409H, L174P, Q497R and V460M, were also investigated in several studies, but rarely showed positive outcomes.

**Table 1:** Characteristics of the studies included in the meta-analysis and the GBA mutations examined.

Author	Year	Region	Number of subjects (*n*)		Mutations examined
			Cases	Controls	
Tan [14]	2007	Singapore^a^	331	347	L444P, N370S
Wu [15]	2007	Taiwan	518	339	L444P, R120W, Rec*Nci*I
Ziegler [16]	2007	Taiwan	92	92	L444P, D409H, L174P, Q497R, N370S, V460M
Sidransky^b^ [17]	2009	Taiwan	183	88	L444P, N370S, R120W, Rec*Nci*I
Hu [18]	2010	Guangdong	328	300	N370S
Mao [19]	2010	Sichuan	616	411	L444P
Huang [20]	2011	Taiwan	967	780	L444P, R120W, D409H, L174P, Q497R
Wang [21]	2012	Hubei	208	298	L444P, N370S, R120W
Zhang [22]	2012	Zhejiang	195	443	L444P, N370S, R120W

a Chinese population recruited in Singapore;

b Data extracted from only the Chinese subgroup.

**Table 2:** Distribution of the studied GBA mutations included in the meta-analysis.

Study	Results of mutations in studies	
	Case (n = 125)	Control (n = 15)
Tan 2007^a+c^	L444P (8), N370S (0)	L444P (0), N370S (0)
Wu 2007^c^	L444P (13), Rec*Nci*I (2), R120W (1)	L444P (2), Rec*Nci*I (2), R120W (0)
Ziegler 2007^c^	L444P (1), N370S (0), D409H (1), L174P (1), Q497R (1), V460M (0)	L444P (0), N370S (0), D409H (0), L174P (0), Q497R (0), V460M (1)
Sidransky 2009^a/b/c^	No details (22)	No details (4)
Hu 2010^b^	N370S (6)	N370S (2)
Mao 2010^b^	L444P (20)	L444P (1)
Huang 2011^b+c^	L444P (27), Rec*Nci*I (7), R120W (0), D409H (2), Q497R (0)	L444P (1), Rec*Nci*I (1), R120W (0), D409H (0), Q497R (0)
Wang 2012^b+c^	L444P (7), N370S (0), R120W (0)	L444P (1), N370S (0), R120W (0)
Zhang 2012^c^	L444P (6), N370S (0), R120W (0)	L444P (0), N370S (0), R120W (0)

a Fluorescence PCR;

b PCR-restriction fragment length polymorphism (RFLP) analysis;

c Sequencing.

### Quantitative data synthesis

**Figure 2:**
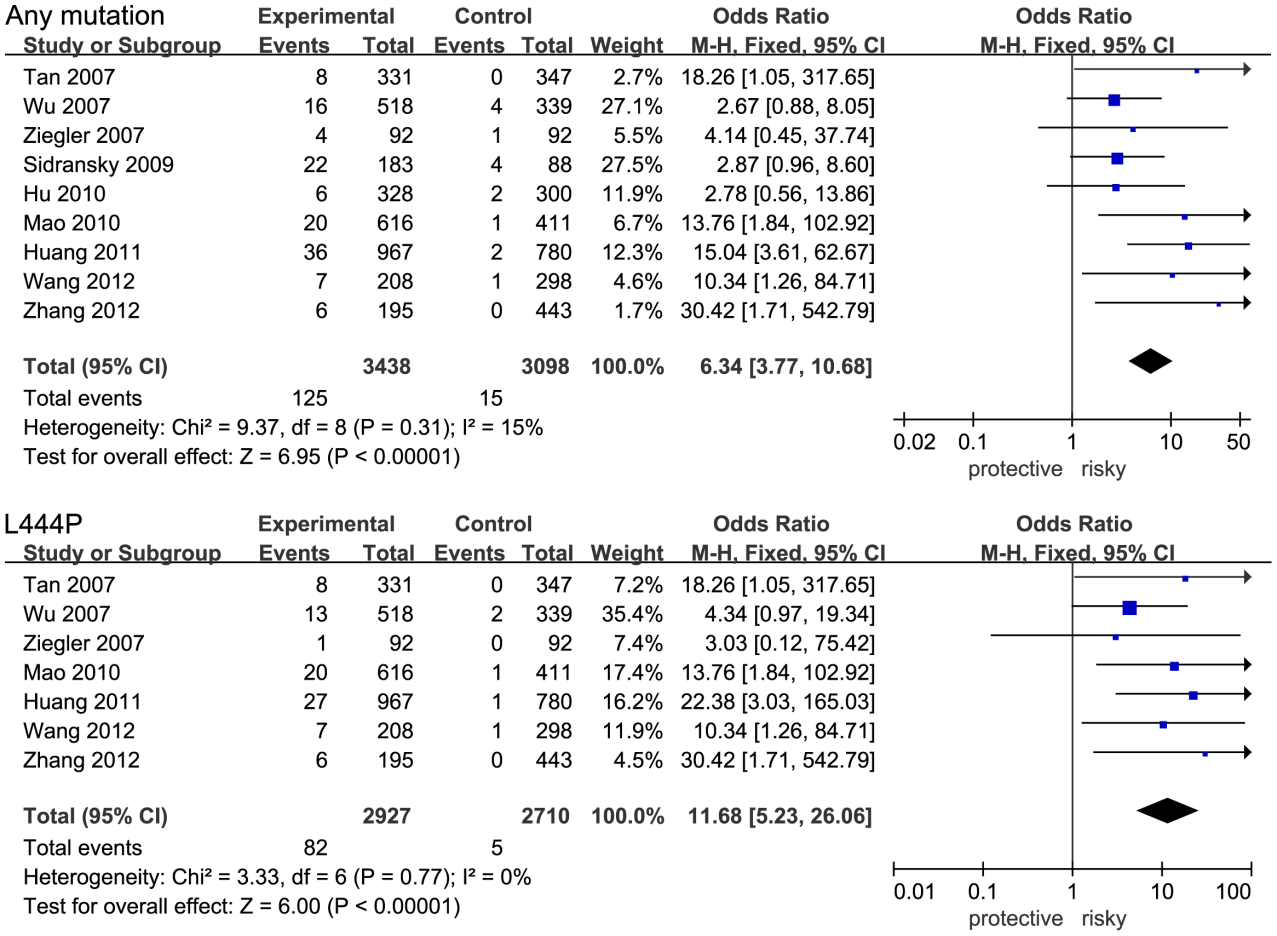
Forest plots of the association between GBA mutations and PD risk in the Chinese population. For the group showing any mutations, OR = 6.34, 95% CI = 3.77–10.68, p<0.00001; for the subgroup with the L444P mutation, OR = 11.68, 95% CI = 5.23–26.06, p<0.00001.

In the current meta-analysis, the associations between any GBA mutation and PD within a Chinese population were evaluated. Among the studies included, no significant difference in heterogeneity was observed (χ^2^ = 9.37, *I* ^2^ = 15%, p = 0.31); thus a fixed effect model was used to calculate the pooled ORs and 95% CIs. The results of the statistical analysis revealed a significant difference in the GBA mutation-related susceptibility (pooled OR = 6.34, 95% CI = 3.77–10.68, p < 0.00001, Fig. 2). A subgroup analysis was performed to investigate the roles of some of the frequent mutations. In the subgroup with the L444P mutation, a significant association with PD was observed (OR = 11.68, 95% CI = 5.23–26.06, p < 0.00001, Fig. 2). No such association was observed for the subgroup with the N370S mutation and other mutations, in part because of the small sample size or rare outcomes. For the low mutation rate and rare positive events, it was hardly to draw a reliable conclusion from quantitative analysis with a high power. The distribution of the rare GBA mutation loci that were studied in Chinese population was shown in Table 2.

## Discussion

Glucocerebrosidase, an enzyme located in lysosome and involved in glycolipid metabolism, has been well known for its role in Gaucher disease (GD). Intriguingly, mutations of GBA gene have been expected to confer a susceptibility to Parkinson's disease, which shows a high rate of comorbidity with GD [28], specifically in Ashkenazi Jewish cohort [29], [30]. GBA mutations, albeit rarer in non-Ashkenazi Jewish, are supposed to play such a role in PD.

Several studies have examined the relationship between GBA mutations and PD risk in Chinese population. To draw a comprehensive understanding, we pooled the data, performed a meta-analysis and reported the distribution of the studied GBA mutations in Chinese population. The meta-analysis included 6536 subjects, with 3438 cases and 3098 healthy controls; however, only 125 mutations in GBA cases (any mutation in cases, 3.64%) and 15 in controls (any mutation in controls, 0.48%) were detected. These results suggested a low rate of GBA mutation in Chinese population (2.14% for overall subjects). The mutation rate of GBA in patients from the Chinese population (3.64%) was also lower than that in patients from Ashkenazi Jews cohort(19.61%) and non-Ashkenazi Jews (6.85%, average of different ancestries) [22]. Nevertheless, a significant association of GBA mutations with PD was obtained after pooling the data. We also examined the role of a few specific mutations, such as L444P and N370S. Unlike N370S, the most common GBA mutation in Ashkenazi Jews (71.89%) [22], L444P, in our study, appeared to be the most mutation in Chinese population (62.14%), exceeding the N370S mutation (5.71%). Statistically, we found a significant relationship of PD with L444P mutation, but not with N370S mutation. Although N370S was one of most common mutations in GBA gene in majority of previous studies, this mutation rarely occurred in Han population, and it was difficult to draw a conclusive result with rare positive outcomes from small sample sizes. Thus, studies with larger sample size are needed to investigate the role of N370S and other rare GBA mutations. Other mutation loci, such as Rec*Nci*I, R120W, D409H, L174P, Q497R and V460M, were also investigated in several studies. However, almost no positive mutation was detected in the majority of these studies, which likely due to their small sample sizes, which brought low statistical powers and less reliable results.

Although primary results of the present retrospective analysis are compelling, this study has a few limitations. First, data included in the meta-analysis were obtained from published articles. We could not track unpublished articles, influencing the comprehensiveness of the data. Second, a language bias might have occurred as some articles published in languages other than English that might have been missed out. Third, the sample size in some of the studies included in our analysis was too small to enhance the power of the meta-analysis. Finally, within-study confounding factors, such as genetic background, might have existed. The Chinese comprise multinational populations with probable genetic differences. In addition, the Chinese subjects recruited in Singapore may have introduced confounding genetic factors.

Taken together, our meta-analysis showed a significant association between GBA mutations and the susceptibility to Parkinson's disease in a Chinese population. Specifically, we demonstrated that the L444P mutation was a positive risk factor in PD. Another common mutation, N370S, failed to produce a significant association with PD, likely due, at least in part, to the small sample size. Thus, additional studies examining the association of GBA mutations, especially N370S and other rare mutations, with PD risk in the Chinese population with large sample sizes are needed.

## Supporting Information

S1 Table **Statistical power for each GBA mutation locus.**
(DOCX)

S1 Checklist **PRISM 2009 checklist.**
(DOC)

S1 File **PRISM 2009 flow diagram.**
(DOC)

## References

[1] Lees AJ, Hardy J, Revesz T (2009) Parkinson's disease. Lancet 373:2055–2066. 19524782 10.1016/S0140-6736(09)60492-X

[2] Trinh J, Farrer M (2013) Advances in the genetics of Parkinson disease. Nat Rev Neurol 9:445–454. 23857047 10.1038/nrneurol.2013.132

[3] Barneveld RA, Keijzer W, Tegelaers FP, Ginns EI, Geurts van Kessel A, et al (1983) Assignment of the gene coding for human beta-glucocerebrosidase to the region q21–q31 of chromosome 1 using monoclonal antibodies. Hum Genet 64:227–231. 6885065 10.1007/BF00279398

[4] Aharon-Peretz J, Rosenbaum H, Gershoni-Baruch R (2004) Mutations in the glucocerebrosidase gene and Parkinson's disease in Ashkenazi Jews. N Engl J Med 351:1972–1977. 15525722 10.1056/NEJMoa033277

[5] Toft M, Pielsticker L, Ross OA, Aasly JO, Farrer MJ (2006) Glucocerebrosidase gene mutations and Parkinson disease in the Norwegian population. Neurology 66:415–417. 16476943 10.1212/01.wnl.0000196492.80676.7c

[6] Nishioka K, Vilarino-Guell C, Cobb SA, Kachergus JM, Ross OA, et al (2010) Glucocerebrosidase mutations are not a common risk factor for Parkinson disease in North Africa. Neurosci Lett 477:57–60. 19945510 10.1016/j.neulet.2009.11.066 PMC2970621

[7] Lwin A, Orvisky E, Goker-Alpan O, LaMarca ME, Sidransky E (2004) Glucocerebrosidase mutations in subjects with parkinsonism. Mol Genet Metab 81:70–73. 14728994 10.1016/j.ymgme.2003.11.004

[8] Eblan MJ, Walker JM, Sidransky E (2005) The glucocerebrosidase gene and Parkinson's disease in Ashkenazi Jews. N Engl J Med 352:728–731. 10.1056/NEJM200502173520719 15716572

[9] Velayati A, Yu WH, Sidransky E (2010) The role of glucocerebrosidase mutations in Parkinson disease and Lewy body disorders. Curr Neurol and Neurosci Rep 10:190–198. 10.1007/s11910-010-0102-x PMC3529411 20425034

[10] Bras J, Paisan-Ruiz C, Guerreiro R, Ribeiro MH, Morgadinho A, et al (2009) Complete screening for glucocerebrosidase mutations in Parkinson disease patients from Portugal. Neurobiol Aging 30:1515–1517. 18160183 10.1016/j.neurobiolaging.2007.11.016 PMC2736795

[11] Socal MP, Bock H, Michelin-Tirelli K, Hilbig A, Saraiva-Pereira ML, et al (2009) Parkinson's disease and the heterozygous state for glucocerebrosidase mutations among Brazilians. Parkinsonism Relat Disord 15:76–78. 18358758 10.1016/j.parkreldis.2008.01.019

[12] Noreau A, Riviere JB, Diab S, Dion PA, Panisset M, et al (2011) Glucocerebrosidase mutations in a French-Canadian Parkinson's disease cohort. Can J Neurol Sci 38:772–773. 21856586 10.1017/s0317167100012300

[13] Choi JM, Kim WC, Lyoo CH, Kang SY, Lee PH, et al (2012) Association of mutations in the glucocerebrosidase gene with Parkinson disease in a Korean population. Neurosci Lett 514:12–15. 22387070 10.1016/j.neulet.2012.02.035

[14] Gibb WR, Lees AJ (1988) The relevance of the Lewy body to the pathogenesis of idiopathic Parkinson's disease. J Neurol Neurosurg Psychiatry 51:745–752. 2841426 10.1136/jnnp.51.6.745 PMC1033142

[15] Guo SW, Thompson EA (1992) Performing the exact test of Hardy-Weinberg proportion for multiple alleles. Biometrics 48:361–372. 1637966

[16] Mantel N, Haenszel W (1959) Statistical aspects of the analysis of data from retrospective studies of disease. J Natl Cancer Inst 22:719–748. 13655060

[17] DerSimonian R, Laird N (1986) Meta-analysis in clinical trials. Control Clin Trials 7:177–188. 3802833 10.1016/0197-2456(86)90046-2

[18] Sim J, Wright CC (2005) The kappa statistic in reliability studies: use, interpretation, and sample size requirements. Phys Ther 85:257–268. 15733050

[19] Tan EK, Tong J, Fook-Chong S, Yih Y, Wong MC, et al (2007) Glucocerebrosidase mutations and risk of Parkinson disease in Chinese patients. Arch Neurol 64:1056–1058. 17620502 10.1001/archneur.64.7.1056

[20] Wu YR, Chen CM, Chao CY, Ro LS, Lyu RK, et al (2007) Glucocerebrosidase gene mutation is a risk factor for early onset of Parkinson disease among Taiwanese. J Neurol Neurosurg Psychiatry 78:977–979. 17702778 10.1136/jnnp.2006.105940 PMC2117856

[21] Ziegler SG, Eblan MJ, Gutti U, Hruska KS, Stubblefield BK, et al (2007) Glucocerebrosidase mutations in Chinese subjects from Taiwan with sporadic Parkinson disease. Mol Genet Metab 91:195–200. 17462935 10.1016/j.ymgme.2007.03.004 PMC1950300

[22] Sidransky E, Nalls MA, Aasly JO, Aharon-Peretz J, Annesi G, et al (2009) Multicenter analysis of glucocerebrosidase mutations in Parkinson's disease. N Engl J Med 361:1651–1661. 19846850 10.1056/NEJMoa0901281 PMC2856322

[23] Hu FY, Xi J, Guo J, Yu LH, Liu L, et al (2010) Association of the glucocerebrosidase N370S allele with Parkinson's disease in two separate Chinese Han populations of mainland China. Eur J Neurol 17:1476–1478. 20528910 10.1111/j.1468-1331.2010.03097.x

[24] Mao XY, Burgunder JM, Zhang ZJ, An XK, Zhang JH, et al (2010) Association between GBA L444P mutation and sporadic Parkinson's disease from Mainland China. Neurosci Lett 469:256–259. 20004703 10.1016/j.neulet.2009.12.007

[25] Huang CL, Wu-Chou YH, Lai SC, Chang HC, Yeh TH, et al (2011) Contribution of glucocerebrosidase mutation in a large cohort of sporadic Parkinson's disease in Taiwan. Eur J Neurol 18:1227–1232. 21338444 10.1111/j.1468-1331.2011.03362.x

[26] Wang Y, Liu L, Xiong J, Zhang X, Chen Z, et al (2012) Glucocerebrosidase L444P mutation confers genetic risk for Parkinson's disease in central China. Behav Brain Funct 8:57. 23227814 10.1186/1744-9081-8-57 PMC3538614

[27] Zhang X, Bao QQ, Zhuang XS, Gan SR, Zhao D, et al (2012) Association of Common Variants in the Glucocerebrosidase Gene with High Susceptibility to Parkinson's Disease among Chinese. Chin J Physiol 55:398–404. 23286447 10.4077/CJP.2011.AMM076

[28] Rogaeva E, Hardy J (2008) Gaucher and Parkinson diseases: Unexpectedly related. Neurology 70:2272–2273. 18541881 10.1212/01.wnl.0000314657.92762.0f

[29] Hruska KS, LaMarca ME, Scott CR, Sidransky E (2008) Gaucher disease: mutation and polymorphism spectrum in the glucocerebrosidase gene (GBA). Hum Mutat 29:567–583. 18338393 10.1002/humu.20676

[30] Beutler E, Nguyen NJ, Henneberger MW, Smolec JM, McPherson RA, et al (1993) Gaucher disease: gene frequencies in the Ashkenazi Jewish population,. Am J Hum Genet 52:85–88. 8434610 PMC1682129

