# Supplementary material for: Glucocerebrosidase Gene Mutations Associated with Parkinson's Disease: A Meta-Analysis in a Chinese population
Source: PLoS One. 2014 Dec 23;9(12):e115747. doi: 10.1371/journal.pone.0115747 (PMC4275276; doi:10.1371/journal.pone.0115747)
Supplement: S1 Table — Statistical power for each GBA mutation locus. (DOCX) [file pone.0115747.s001.docx]

Table S1. Statistical power for each GBA mutation locus

| Mutaiton | Studies included | Events in cases | Events in controls | Odds ratio[95%CI] | Statistical power |
| --- | --- | --- | --- | --- | --- |
| L444P | 8 | 82(2927) | 5(2710) | 11.68[5.23,26.06] | 100% |
| N370S | 5 | 6(1154) | 2(1480) | 2.78[0.56,13.86] | 42.89% |
| RecNciI | 2 | 9(1485) | 3(1119) | 1.87[0.22,16.20] | 24.28% |
| R120W | 4 | 1(1888) | 0(1860) | 1.97[0.08,48.45] | 16.83% |
| D409H | 2 | 3(1059) | 0(872) | 3.57[0.39,32.30] | 34.96% |
| L174P | 1 | 1(92) | 0(92) | 3.03[0.12,75.42] | 17.08% |
| Q497R | 2 | 1(1059) | 0(872) | 3.03[0.12,75.42] | 14.84% |
| V460M | 1 | 0(92) | 1(92) | 0.33[0.01,8.20] | 17.08% |

PASS 11.0 software was used for calculation of power values; α=0.05
