# Supplementary material for: Glucocerebrosidase Gene Mutations Associated with Parkinson's Disease: A Meta-Analysis in a Chinese population
Source: PLoS One. 2014 Dec 23;9(12):e115747. doi: 10.1371/journal.pone.0115747 (PMC4275276; doi:10.1371/journal.pone.0115747)
Supplement: S1 Checklist — PRISM 2009 checklist. (DOC) [file pone.0115747.s002.doc]

| **Section/topic** | **#** | **Checklist item** | **Reported on page #** |
| --- | --- | --- | --- |
| **TITLE** | | |  |
| Title | 1 | Glucocerebrosidase gene mutations associated with Parkinson’s disease: A meta-analysis in a Chinese population | 1 |
| **ABSTRACT** | | |  |
| Structured summary | 2 | **Background:** Mutations of glucocerebrosidase (GBA) confer susceptibility to Parkinson’s disease in several ethnical populations, with an especially high incidence in the Ashkenazi Jewish population. Although studies have investigated a similar association in Chinese populations, small sample sizes and few positive outcomes have made it difficult to obtain conclusive results from these individual studies.  **Methodology/Principal Findings:** Systematic searches of electronic databases Embase, PubMed, Cochrane library and PDgene, as well as hand searching of the references of identiﬁed articles were performed. Statistical analyses were performed using software Review Manager (Version 5.2). The pooled odds ratios (ORs) with 95% confidence intervals (95%CIs) were calculated. Fixed or random effects model was separately used depending on the heterogeneity between studies. A total of 9 studies including 3814 cases and 3387 controls were combined showing significant statistical evidence of association between overall GBA mutations and PD (OR=6.63, 95%CI=3.94-11.15, p<0.00001). For L444P subgroup (OR=11.08, 95%CI=5.06-24.28, p<0.00001). No significance was observed in N370S subgroup, partly because of small sample size.  **Conclusions/Significance:** This meta-analysis indicates that GBA mutations are associated with PD patients among Chinese population. The mutations of L444P and N370S are most studied and related with Chinese. However, this conclusion should be interpreted with caution due to the small sample size. Larger sample-size studies with more mutation loci, as well as homogeneous PD patients and well-matched controls, are required. | 2 |
| **INTRODUCTION** | | |  |
| Rationale | 3 | Parkinson disease (PD), which ranks second only to Alzheimer’s disease among neurodegenerative disorders, is pathologically characterized by the degeneration of dopaminergic neurons in the nigro-striatal system and clinically characterized by insidiously progressive movement impairments, such as rigidity, bradykinesia, impaired balance and tremor at rest. PD is an age-dependent disease, and the prevalence among aged people (more than 65 years old) reaches 1.8 % in Europe and 1.7 % in China. Besides aging and environmental factors, genetic risk is also considered as a critical factor for PD, including SNCA, LRRK2, MAPT and other genetic loci, which had been identified as susceptibility genes for familial and common sporadic forms of PD. Mutations of glucocerebrosidase (GBA) had been reported to be significantly associated with PD susceptibility in patients of Ashkenazi Jewish, which was reported with a high mutation rate of GBA gene. Subsequently, evidence of this relationship was provided by several studied in non-Ashkenazi Jewish populations, although there were also some studies with negative results. Among all these GBA mutations, the heterozygotes for L444P and N370S revealed to be the most common variants, and patients with PD were more than five times likely to carry the mutations than those without PD. | 2-3 |
| Objectives | 4 | The present review was therefore designed to derive a more precise estimation of the distribution and association between GBA mutations and PD. | 3 |
| **METHODS** | | |  |
| Protocol and registration | 5 | No protocol and registration. |  |
| Eligibility criteria | 6 | (1) studies on association between GBA mutations and PD among Chinese population; (2) articles with full text; (3) sufficient data to calculate the odd ratio (OR), confidence interval (CI) and p value; (4) specified genotyping methods or provided appropriate references; and (5) diagnoses of PD patients had to fulfill the criteria of U.K. Parkinson’s Brain Bank. | 4 |
| Information sources | 7 | PubMed, Embase, Cochrane library and PDgene database were searched from the first available year to February 20, 2014, as well as hand searching of the references of identiﬁed articles were performed. | 3 |
| Search | 8 | Search strategy: “glucocerebrosidase” or “GBA”, “polymorphism” or “variant” or “mutation”, “parkinson’s disease” or “PD” and “Chinese”. | 3 |
| Study selection | 9 | Two investigators (Chen J and Li W) screened each of the titles, abstracts, and full texts to determine inclusion independently. The results were compared and disagreements were resolved by consensus. | 4 |
| Data collection process | 10 | Information was carefully extracted from all included publications independently by two of the authors (Chen J and Li W) according to the inclusion criteria listed above. Disagreement was resolved by consensus. If these two authors could not reach a consensus, another author (Zhang T) was consulted. | 4 |
| Data items | 11 | The following data were collected from each study: first author’s name, publication date, region, mutation loci, diagnoses of PD, total numbers of cases and controls, frequency of GBA mutations in cases and controls or published crude odds ratios (ORs) derived from these data. | 4 |
| Risk of bias in individual studies | 12 | The quality of included studies was evaluated independently by two authors (Chen J and Li W) of this article. Inter-rate reliability was used to make sure the selection qulity. | 4 |
| Summary measures | 13 | The principal summary measures are odds ratios (ORs) and 95% confidence intervals (CIs). | 4 |
| Synthesis of results | 14 | The strength of association between GBA mutations and PD risk was measured by ORs and 95%CIs. The combined ORs were calculated for overall GBA mutations and specified mutation respectively. We used the generic inverse variance method to obtain pooled ORs, weighting each study by the inverse of the square of the standard error of its study-specific OR. The ORs were pooled through a fixed effects model when no heterogeneity was observed among studies. Otherwise, a random effects model was adopted. | 4 |

Page 1 of 2

| **Section/topic** | **#** | **Checklist item** | **Reported on page #** |
| --- | --- | --- | --- |
| Risk of bias across studies | 15 | An estimate of potential publication bias was not performed because of the limited number of studies we included. | 4 |
| Additional analyses | 16 | Subgroup analyses were performed by most frequent specified mutation. | 4 |
| **RESULTS** | | |  |
| Study selection | 17 | Based on our preliminary search criteria, a total of twelve publications were eligible. Among these articles, one study had no healthy controls, two studies had no sufficient data and could not retrieve after contacting author. Hence, nine studies were included in the final analysis, including 3814 cases and 3387 controls. | 5 |
| Study characteristics | 18 | Table 1 shows the studies included in the meta-analysis and their main characteristics. Table 2 shows the distribution of related mutations. | 11 |
| Risk of bias within studies | 19 | All studies we had included were published on peer-review publications. Meanwhile, the NOS was referred to when we screened records, to make sure of the high methodological quality. | 5 |
| Results of individual studies | 20 | The main results of individual studies were shown in Figure 2. |  |
| Synthesis of results | 21 | There was significant association between overall GBA mutations and PD risk (OR = 6.63, 95% CI = 3.94-11.15, p＜0.00001), and for L444P subgroup (OR = 11.08, 95% CI = 5.06-24.28, p ＜ 0.00001). No significance was observed in N370S subgroup. | 5 |
| Risk of bias across studies | 22 | Sensitivity analysis was not performed because of limited heterogeneity. As well, because of limited number of studies we included, publication bias was not performed. |  |
| Additional analysis | 23 | **Subgroup analysis:** Association between specified GBA mutation and PD risk was estimated. For L444P subgroup (OR = 11.08, 95% CI = 5.06-24.28, p ＜ 0.00001). No significance was observed in N370S subgroup. Other mutation loci, like Rec*Nci*I, R120W, D409H, L174P, Q497R and V460M, were also investigated in several studies, however, there were almost no positive mutations detected in majority of studies, probably due to their small samples. | 5 |
| **DISCUSSION** | | |  |
| Summary of evidence | 24 | A total of nine publications were included in our meta-analysis. Our meta-analyses showed statistical evidence for association between GBA mutations and PD among Chinese population, especially the L444P mutation. | 5 |
| Limitations | 25 | **Limitations:** Firstly, Studies included in the present study were from published articles. We could not track all unpublished articles to obtain data for analysis; Secondly, Language bias might have occurred, for some articles published in non-English could been missed; Thirdly, the sample size of some studies included in our analysis are not big enough to enhance the power of the meta-analysis; Finally, other inner confounding factors may also exist. | 6-7 |
| Conclusions | 26 | **Conclusion：**Taken together, our pooled analysis provides a significant association between GBA mutations and Parkinson’s disease susceptibility among Chinese population. Specifically, L444P mutation is demonstrated to be a positive factor in PD risk. Another common mutation, N370S, fails to produce significant result, partly for the reason of the small sample size. Thus, large sample studies interested in GBA mutations, specially N370S and other rare mutations with PD risk among Chinese population are needed. | 7 |
| **FUNDING** | | |  |
| Funding | 27 | This study was supported by Grants from the National Natural Science Foundation of China (81271411) & Natural Science Foundation Project of CQ CSTC (cstc2012jjA10098). | 7 |

*From:*  Moher D, Liberati A, Tetzlaff J, Altman DG, The PRISMA Group (2009). Preferred Reporting Items for Systematic Reviews and Meta-Analyses: The PRISMA Statement. PLoS Med 6(6): e1000097. doi:10.1371/journal.pmed1000097

For more information, visit: **www.prisma-statement.org**.

Page 2 of 2
