# Supplementary material for: Glucocerebrosidase Gene Mutations Associated with Parkinson's Disease: A Meta-Analysis in a Chinese population
Source: PLoS One. 2014 Dec 23;9(12):e115747. doi: 10.1371/journal.pone.0115747 (PMC4275276; doi:10.1371/journal.pone.0115747)
Supplement: S1 File — PRISM 2009 flow diagram. (DOC) [file pone.0115747.s003.doc]

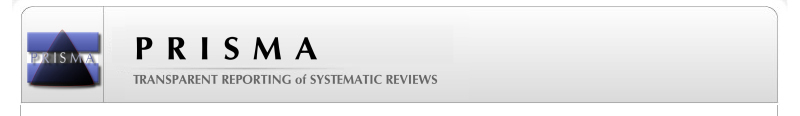
**PRISMA 2009 Flow Diagram**

**Screening**

**Included**

**Eligibility**

**Identification**

Records identified through database searching
(n = 82)

Additional records identified through other sources
(n = 0)

Records after duplicates removed
(n = 65)

Records screened
(n = 23)

Records excluded
(n = 42)

Full-text articles assessed for eligibility
(n = 9)

Full-text articles excluded, with reasons of repetitive studies and insufficient data (n = 14)

Studies included in qualitative synthesis
(n = 9)

Studies included in quantitative synthesis (meta-analysis)
(n = 9)
